# Clinical and ultrasonographic findings of some ocular conditions in sheep and goats

**Published:** 2013-01-23

**Authors:** O. El-Tookhy, M. Tharwat

**Affiliations:** 1 *Department of Surgery, Faculty of Veterinary Medicine, Cairo University, Egypt*; 2 *Department of Animal Medicine, Faculty of Veterinary Medicine, Zagazig University, Egypt*

**Keywords:** Eye, Goat, Ocular, Sheep, Ultrasound

## Abstract

This study was carried out to describe the ultrasonographic findings in relation to the clinical symptoms of some common ocular conditions in sheep and goats. Fifty animals (32 goats and 18 sheep) with different ocular problems were examined. Ultrasonographic examination was performed using a B-mode ocular ultrasound unit, and the structure of the globe was evaluated at a depth of 4-6 cm. Early cases (*n*=35, 70%) showed varying ocular conditions; hypopyon, (*n*=8, 16%), stromal abscesses, (*n*=4, 8%), and anterior uveitis (*n*=23, 46%). Hypopyon appeared clinically as a white or yellowish material in the anterior chamber, and ultrasonographically as a hyperechoic mass in the anterior chamber. Severe iridocyclitis was noticed in acute cases of infectious keratoconjunctivitis (IKC) accompanied by blepharospasm, photophobia, excessive tearing and eyelid margin crust formation. Ultrasonographically, the pupil appeared constricted with increased hyperechoic thickening of the ciliary body. In chronic cases of IKC, corneal pigmentation (*n*=5, 10%) and cataract (*n*=10, 20%) were seen. Ultrasonographically the type and degree of cataract were diagnosed. The present study provides an inside view of the inner ocular structures during the course of certain eye diseases where ophthalmoscopic examination is not possible. Our findings, although preliminary, are relevant for the more complete diagnosis of certain external ocular conditions in sheep and goat herds.

## Introduction

It is known that ocular diseases in food-producing animals play a significant role in economic losses (Whittaker *et al.*, 1999; Waldridge and Colitz 2002; Smith and Sherman, 2009). In sheep and goats, outbreaks of infectious keratoconjunctivitis (IKC) and cases of phenothiazine toxicosis may cause corneal opacity (Whittaker *et al.*, 1999; Waldridge and Colitz, 2002).

Corneal edema is a common clinical sign of corneal ulceration, keratitis, anterior uveitis, and many systemic diseases, and precludes the direct visualization of intraocular structures by ophthalmoscopy (Whittaker *et al.*, 1999). Under such conditions; alternative diagnostic methods for intraocular diseases must be explored (Boroffka *et al.*, 1998; Bentley *et al.*, 2003; Scotty *et al.*, 2004).

Ultrasonography is a useful tool to evaluate the contents of the globe and orbit as is done routinely in companion animal medicine (Ribeiro *et al.*, 2009). Transcorneal ultrasonography enables the evaluation of intraocular structures in eyes with opaque, diseased corneas in order to evaluate the prognosis for vision following resolution of the corneal disease.

Indications for ocular ultrasound include any clinical entity which impedes visualization of the globe and retrobulbar region. Severe corneal edema, corneal lacerations or ulcerations, cataracts or ocular masses may preclude visualization of deeper structures with traditional ophthalmoscopic methods. Another common indication for ocular ultrasound is disparity in globe size or an exophthalmic globe.

Ultrasound is important to differentiate between enophthalmos, buphthalmus, or exophthalmus due to the presence of retrobulbar masses (Whitcomb, 2002). Knowledge of the ultrasonographic appearance and normal dimensions of the eye would serve as a basis for ultrasonographic examinations when ocular disease may have caused alterations in the dimensions and appearance intraocular structures (Potter *et al.*, 2008). Ultrasonography of sheep and goats with ocular diseases are not widely reported, therefore, this study attempted to report the ultrasonographic findings in relation to the clinical symptoms of the common ocular conditions in sheep and goats.

## Materials and Methods

### Animals and clinical examination

Fifty animals (32 goats and 18 sheep) at an average age of 22.0±8.5 months and weight of 40.8±12 kg were included in the study. All animals were examined at the Veterinary Teaching Hospital, Qassim University, Saudi Arabia (January, 2010-June, 2012) for ocular complaints in one or both eyes. Case histories were recorded based on their owners’ knowledge. Each animal received a full clinical examination including general behavior and condition, auscultation of the heart, lungs, rumen and intestine, detection of heart and respiratory rates, and rectal temperature measurement. Regular ophthalmic examination included ophthalmoscopy and tonometry. This was followed by ultrasonographic examination. All animals were handled according to the *Laboratory Animal Control Guidelines*, which basically conform to the *Guide for the Care and Use of Laboratory Animals* of the National Institutes of Health in the USA (NIH publications No. 86 to 23, revised 1996).

### Ultrasonographic examination

For ocular sedation, surface corneal anesthetic 2% Lidocaine (Norbrook Laboratories, UK) was used (Shah, *et al.*, 2010). The animal head was firmly held, tilted and the eyelids were spaced out. Ultrasound coupling gel was applied to the cornea. Ultrasonographic examination was performed using a B-mode ocular ultrasound unit (Aloka SSD-500, Tokyo, Japan) equipped with a 7.5 MHz sector transducer.

The structures of the globe were evaluated to a depth of 4–6 cm. The retrobulbar region was evaluated at a scanning depth of 6–10 cm (Whitcomb, 2002). Ocular abnormalities were evaluated in respect to location (i.e., anterior/posterior segment) and echo texture (isoechoic or hypo/hyper-echoic) compared to the surrounding tissues.

Using the direct corneal contact technique, the transducer was placed directly on the cornea after spreading the coupling gel. Light pressure was applied to maintain good contact between the transducer and the cornea. Each eye was examined initially in horizontal section with the ultrasound beam running from the medial to the lateral canthi, and then the head of the transducer was rotated 90° to visualize the vertical section of the eye.

After ultrasound examination, excess coupling gel was carefully wiped from the eyes. All images were digitally recorded and those views analyzed which gave the maximum information.

## Results

In this study, IKC was the most common ocular affection in sheep and goats. It was reported in 23 cases (5 sheep and 18 goats) whereas cataract was seen in 10 cases (5 sheep and 5 goats). Hypopyon was noticed in 8 cases (6 sheep and 2 goats). Pigmentary keratitis was found in 5 cases (1 sheep and 4 goats) whereas stromal abscesses were noted in 4 cases (1 sheep and 3 goats). Data collected is shown in Table (1).

**Table (1):** Different ocular conditions seen in sheep and goats.

	Acute conditions (n=35, 70%)			Chronic conditions (n=15, 30%)	
	IKC	Stromal Abscess	Hypopyon	Pigmentary Keratitis	Cataract
Sheep	5	1	6	1	5
Goats	18	3	2	4	5
Total	23	4	8	5	10
%	46	8	16	10	20

**Fig 1:**
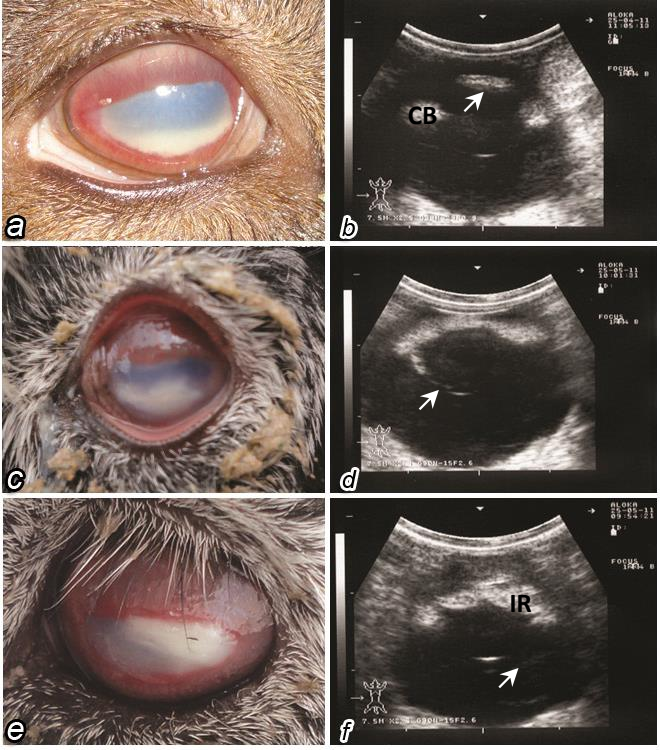
***a***-conjunctival injection with corneal opacity and anterior chamber hypopyon, ***b***-hypopyon seen as hyperechoic shadow in the anterior chamber (*arrow*) with thickening of the ciliary body (CB) and increased in its echogenicity, ***c***-keratitis, conjunctivitis, corneal ulceration and hypopyon, ***d***-sever iritis with slight posterior capsular cataract (*arrow*), ***e***-sever conjunctivitis, corneal stromal abscess (*arrow*) and dryness, ***f***-iridocyclitis manifested by hyperechoic iris (IR) and ciliary body with vitreal exudates (*arrow*).

The clinical signs of primary keratoconjunctivitis and iridocyclitis were interlaced and could not be distinguished from each other by ultrasound. Clinical signs were blepharospasm, serous ocular discharge, conjunctival hyperemia, corneal neovascularization and cellular infiltration of the anterior stroma; with signs of a reflex uveitis and hypopyon (Fig. 1). Treatment of those conditions consisted mainly of a mix of antibiotic/anti-inflammatory topical ointments together with systemic antibiotics. Topical treatment was with Multiject^®^ eye ointment (procaine penicillin G, streptomycin sulphate, neomycin and prednisolone; Norbrook Laboratories Ltd., UK), together with systemic antibiotic PenStrep^®^ (penicillin streptomycin 40.000 IU/kg BW IM/5d; Norbrook Laboratories, UK). All cases with the exception of 5 animals improved following 5 days of successive treatment.

In acute cases with keratitis and panophthalmitis (*n*=35/50, 70%), specific conditions were seen such as hypopyon, (*n*=8, 16%), stromal abscesses, (*n*=4, 8%), and uveitis (*n*=23, 46%). Hypopyon occurred (Fig. 1a and 1c) and appeared clinically as a white or yellowish material floating in the anterior chamber. Inflammatory and pyogenic materials in the anterior chamber were seen ultrasonographically as a hyperechoic mass in the anterior chamber (Fig. 1b).

Stromal abscesses developed as painful white or yellow areas within the cornea that often did not stain with fluorescein (Fig. 1e). Blood vessels slowly grew into the cornea and associated inflammation with the eye (iritis, uveitis) developed as the corneal disease worsened and contributed to the pain associated with the stromal abscesses. The diagnosis largely based on the history of a corneal injury and the appearance of the lesion. Although medical therapy was often tried initially to treat this condition, many if not most cases progressed despite therapy and ultimately need surgery in order to have any hope of resolving the problem favorably. Excision of the lesion in the stroma enabled tissue to heal better and provided material for culture. Severe iridocyclitis was noticed in acute cases of keratoconjunctivitis accompanied with pain and excessive tearing and crust formation (Fig. 1c). Ultrasonographically, the pupil appeared constricted and hyperechoic with thickening of the ciliary body and increased of its echogenicity (Fig. 1d and 1f). Corneal ulceration and opacity were seen in older cases accompanied with severe degrees of iridocyclitis (Fig. 1e and 1f).

**Fig 2:**
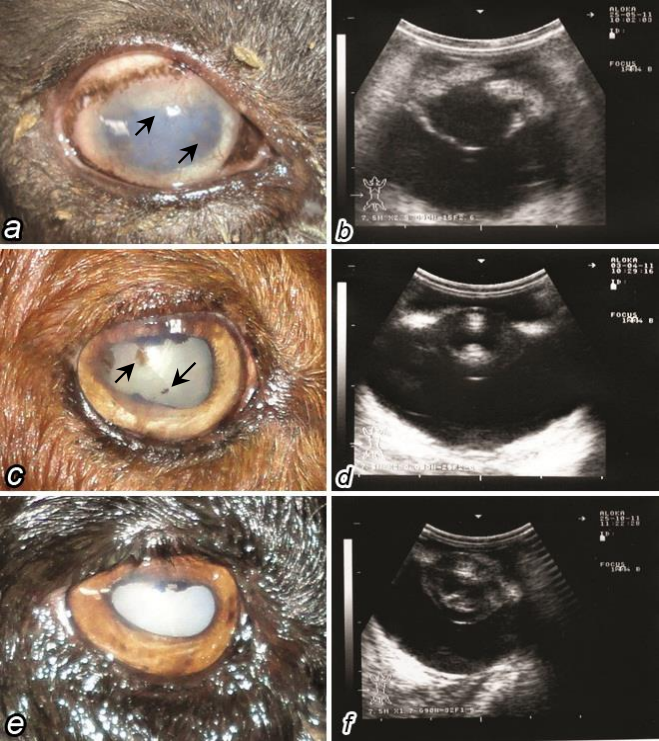
***a***-slight corneal opacity and with neovascularization (*arrows*), and dilated pupil, ***b***-posterior capsular cataract with mild degree of iridocyclitis, ***c***- clear corneal tissue with dilated pupil, corneal pigmentation (*arrows*) as a result of anterior synechia and cataractous lens, ***d***-slight capsular cataract with cortical involvement, ***e***-mature cataract, ***f***-capsular and cortical cataract with slight nuclear involvement.

In more chronic cases (*n*=15/50, 30%), corneal pigmentation (*n*=5, 10%) and cataract (*n*=10, 20%) were seen. Corneal pigmentation took place as a result of pigmentary keratitis with melanin pigment deposited with the corneal stroma (Fig. 2a), or following anterior synechia where a part of the iris adhered to the endothelial corneal layer (Fig. 2b). Cataract of different types and degrees were noted (Fig. 2a, 2c and 2e). Ultrasonographically the type and degree of cataract was evaluated into: posterior capsular (Fig. 2b), anterior and posterior cortical (Fig. 2d) and complete mature cataract (Fig. 2f).

## Discussion

Eyes of goats are frequently used as animal models for diseases of humans (Gilliland *et al.*, 2005; Pawar and Majumdar, 2007; Mohammadi *et al.*, 2011), or for training novice surgeons in different techniques of phacoemulsification for cataract removal (Sudan *et al.*, 2002; Dada and Sindhu, 2000; Mohammadi *et al.*, 2011). Similar to other food-producing animals, ocular diseases in sheep and goat play a significant role in economic losses (Whittaker *et al.*, 1999; Waldridge and Colitz, 2002; Potter *et al.*, 2008), however, individual ophthalmic examinations are not frequently performed as part of a herd health program (Townsend, 2010).

In sheep and goats, corneal edema is a common clinical sign of various ocular conditions such as: corneal ulceration, keratitis, anterior uveitis, and many systemic diseases, and precludes the direct visualization of intraocular structures by ophthalmoscopy (Whittaker *et al.*, 1999). Outbreaks of infectious diseases and cases of toxicosis may also cause corneal opacity (Whittaker *et al.*, 1999; Waldridge and Colitz, 2002). Under such conditions, alternative diagnostic methods forintraocular diseases such as transcorneal ultrasonography must be explored (Boroffka *et al.*, 1998; Bentley *et al.*, 2003 and Scotty *et al.*, 2004). IKC or pinkeye is recognized world-wide as a common condition affecting the eyes of domestic sheep and goats (Giacometti *et al.*, 2002). It is a very painful disease which usually appears during hot dry weather conditions and spreads through close contact and flies. In this study, IKC was diagnosed in 23 animals (5 sheep and 18 goats).

Symptoms of IKC were hyperemia, serous lacrimation, increased blinking and blepharospasm as described by Egwu *et al*. (1989). Temporary blindness was evident in acute cases (n=35). It was common that both eyes become affected, although the clinical signs may have started in one eye only. Later, the conjunctival blood vessels became dilated (conjunctival hyperemia) and migrated across the cornea (corneal vascularisation). During the course of IKC, the exudates were purulent. Results indicated the presence of corneal ulcers in severe, naturally occurring cases of IKC, similar to that described by Hosie and Greig (1995). It was also reported that corneal ulcers do not occur in experimental infections (Trotter *et al.*, 1977). Mycoplasma conjunctivae is the most commonly reported organism from outbreaks of keratoconjunctivitis. It may affect one or both eyes (Giacometti *et al.*, 2002). Experimental infections (Hosie and Greig, 1995) with Mycoplasma conjunctivae as a sole agent did not appear to produce the severe form of ovine keratoconjunctivitis observed in some field infections. This severe form of the disease may be attributable to the presence of other predisposing factors, pyogenic bacteria capable of establishing a secondary super-infection or variations in the sheep’s resistance or the virulence of Mycoplasma conjunctivae. Ocular disease associated with infection by Listeria monocytogenes (Silage eye) has been reported in the veterinary literature for over 30 years (Kummeneje and Mikkelsen, 1975); however it remains a poorly defined condition (Laven and Lawrence, 2006).

Clinical courses include signs of keratoconjunctivitis and uveitis, and cases recover without any residual lesions after antibiotic therapy (Erdogan, 2010). Colesiota conjunctivae, (synonym: Chlamydia psittaci ovis), is a primary cause of contagious kerato-conjunctivitis in sheep, but the clinical picture is complex and is a result of the interaction between the infecting chlamydiae, host resistance factors, and secondary infections caused by opportunistic bacterial ocular pathogens (Bogaard, 1984). Other causative agents are listed such as Staphylococcus aureus, Corynebacterium spp., Escherichia coli and Moraxella (Branhamella) ovis Mycoplasma ovipneumoniae, Acholeplasma oculi and a wide variety of other bacteria (König, 1983; Dagnall, 1994; Åkerstedt and Hofshagen, 2004).

Bacterial infections are usually transient and permanent blindness is rare. Animals, when treatment was appropriately applied, usually begin to recover after a week, but some may remain ill for 3 to 4 weeks with weakness and fever (Åkerstedt and Hofshagen, 2004). Treatment is aimed at reducing the inflammation while determining and eliminating the underlying cause. Prognosis depends on the severity of the condition at the time of treatment and the underlying cause. If the condition is left untreated, glaucoma, lens luxation, and blindness can result. When the cornea is injured microorganisms can be inoculated into the stroma causing stromal abscess. Stromal abscess was seen in 4 animals (1 sheep and 3 goats).

Unlike corneal ulcers, stromal abscess often does not stain with fluorescein because the surface epithelium heals over the injury and the infective organisms are trapped within the cornea where they can elicit an inflammatory response from immune system. White blood cells gain access to the corneal stroma (leukocytic invasion) and release enzymes which break down the stromal tissue between the epithelial and endothelial layers forming the abscess.

This is associated with loss of tissue, tear film proteinase homeostasis, and severe iridocyclitis (Brooks, 2005).

Medical treatment for corneal abscess with antibiotics is practiced but with little value. Surgical management such as posterior lamellar keratoplasty (PLK) and deep lamellar endothelial keratoplasty to treat nonresponsive deep stromal abscesses are conducted with great success but mainly for valuable animals such as horses due to the costs of the procedures (Andrew, 1999; Andrew *et al.*, 2000; Brooks, 2005; Brooks *et al.*, 2008). No surgical attempts were tried in this study due to the costs, also no record of surgical treatment was found on ruminants. Therapeutic regimens that are appropriate for use in ruminants, particularly animals that may be used for meat or milk must be observed (Townsend, 2010).

In this study, hypopyon was noticed in 8 cases (6 sheep and 2 goats). Ultrasonographic imaging showed thickening of the iridial mass and constricted pupil. The ciliary body appeared significantly hyperechoic as a result of inflammation. Inflammatory and pyogenic materials were seen in the anterior chamber as hyperechoic mass. In some cases the lens was affected with different degrees of opacity which was seen as varying hyperechoic shades. It was reported that further infection of the anterior chamber following IKC with/out corneal ulcers rarely leads to hypopyon, panophthalmitis, and shrinking of the ocular globe with permanent blindness (Åkerstedt and Hofshagen, 2004).

Cataract was the second most common problem seen in 10 animals (5 sheep and 5 goats). Naturally, the lens grows throughout life building up layer after layer of fiber cells around a central core and never shed cells. So the cells at the centre of the lens are as old as the animal itself. Some proteins at the centre of the lens may be there for most of the lifetime of the animal. There are no other proteins which persist for so long and alterations occur as consequences of ageing and cataract (Harding and Dilley, 1976).

Cataract can be due to hereditary or acquired causes. In many breeds of sheep and goats it is hereditary and often present at birth (Williams, 2010). Other factors can contribute to the formation of cataracts. These can include metabolic diseases, trauma to the globe, exposure to toxins, and inflammatory diseases such as uveitis. Cataracts linked to genetic defects generally include otherocular abnormalities such as retinal detachment, aniridia, microphakia, andhydrocephalus (Williams, 2010). Such conditions were difficult to find because most animals with a spontaneous idiopathic condition present as a single case in a farm population are normally euthanized and disposed of without even alerting the veterinarian responsible for the flock or herd (Williams, 2010).

An association between cataract and intraocular oxidative stress as determined by the concentration of the protective antioxidant enzymes superoxide dismutase, catalase, and glutathione peroxidase was established (Hässig *et al.*, 2009). Treatment is reserved for cataracts that cause blindness. There is no effective medical therapy for cataracts. Treatment involves surgical removal of the lens but because of the complicated and expensive nature of this surgery, it is not commonly done.

## Conclusion

Individual ophthalmic examinations are not frequently performed as part of a herd health program but are very important to ensure the health of herd. In the examined cases, ocular ultrasonography enabled the visualization of inner ocular structures especially when corneal opacity prevented visualization of retrobulbar region. From the owners perspective ultrasound provided a more precise tool for investigating the eyes and was influential in making decisions regarding the treatment of their animals.

From the practitioners’ point of view, detection of ocular abnormalities using ultrasound prior to any interference helped in planning the possible way(s) of management and treatment. It also provides a better prognosis of the expected outcomes.
